# tRNA gene diversity in the three domains of life

**DOI:** 10.3389/fgene.2014.00142

**Published:** 2014-05-26

**Authors:** Kosuke Fujishima, Akio Kanai

**Affiliations:** ^1^NASA Ames Research CenterMoffett Field, CA, USA; ^2^Institute for Advanced Biosciences, Keio UniversityTsuruoka, Japan

**Keywords:** intron-containing tRNA, split tRNA, permuted tRNA, nev-tRNA, armless tRNA, RNA splicing endonuclease, co-evolution

## Abstract

Transfer RNA (tRNA) is widely known for its key role in decoding mRNA into protein. Despite their necessity and relatively short nucleotide sequences, a large diversity of gene structures and RNA secondary structures of pre-tRNAs and mature tRNAs have recently been discovered in the three domains of life. Growing evidences of disrupted tRNA genes in the genomes of Archaea reveals unique gene structures such as, intron-containing tRNA, split tRNA, and permuted tRNA. Coding sequence for these tRNAs are either separated with introns, fragmented, or permuted at the genome level. Although evolutionary scenario behind the tRNA gene disruption is still unclear, diversity of tRNA structure seems to be co-evolved with their processing enzyme, so-called RNA splicing endonuclease. Metazoan mitochondrial tRNAs (mtRNAs) are known for their unique lack of either one or two arms from the typical tRNA cloverleaf structure, while still maintaining functionality. Recently identified nematode-specific V-arm containing tRNAs (nev-tRNAs) possess long variable arms that are specific to eukaryotic class II tRNA^Ser^ and tRNA^Leu^ but also decode class I tRNA codons. Moreover, many tRNA-like sequences have been found in the genomes of different organisms and viruses. Thus, this review is aimed to cover the latest knowledge on tRNA gene diversity and further recapitulate the evolutionary and biological aspects that caused such uniqueness.

## Introduction

Transfer RNA (tRNA) is a short non-coding RNA of approximately 70–100 bases. The principal function of tRNA is its involvement in translation machinery. Each tRNA is charged with a corresponding amino acid and delivered into the ribosome during protein biosynthesis. Currently all living organism possess tRNA molecules and thus it is known as one of the most classical RNA molecules found in nature, and it is essential for the core biological system. The recent development in the fields of genomics and transcriptomics has revealed many non-canonical tRNA genes and their structures in the three domains of life. These include tRNA gene disruption, fragmentation, rearrangement, minimization, and re-coding (Kanai, 2013). It is likely that such diversification is a consequence of the co-evolution of tRNA and its processing enzyme known as splicing endonuclease (Tocchini-Valentini et al., 2005a). RNA splicing endonuclease in Archaea recognizes and cleaves a structural motif consisting of two three-nucleotide bulge loops separated by four base pairs, known as the bulge-helix-bulge (BHB) motif (Thompson and Daniels, 1990). Structural motif and location of tRNA introns in Archaea have been affected by the change in the recognition and activity of the different unit compositions in the tRNA splicing endonucleases (Fujishima et al., 2011). Some type of disrupted tRNA genes such as split tRNA are considered as potential analogs of early tRNA, thus creating a hot debate in the field of tRNA evolution (Randau and Söll, 2008; Di Giulio, 2012). While it is still unclear how tRNA molecules originated, evolutionary biologists continue to question how these chains of ribonucleotides became involved in the context of protein synthesis, and how they influenced the evolution of these biological systems. When in the course of molecular evolution did tRNA molecule and its characteristic cloverleaf structure emerged is still an ongoing debate, however several evolutionary models representing the origin and convergence of proto-tRNA have been proposed (Weiner and Maizels, 1999; Widmann et al., 2005; Sun and Caetano-Anollés, 2007). In this review, we will recapitulate the characteristics of modern tRNA gene diversity, summarize the coevolutionary scenario of tRNA and their processing enzymes, and provide different models for the origin and evolution of early tRNA.

## tRNA gene diversity in the three domains of life

**Figure 1:**
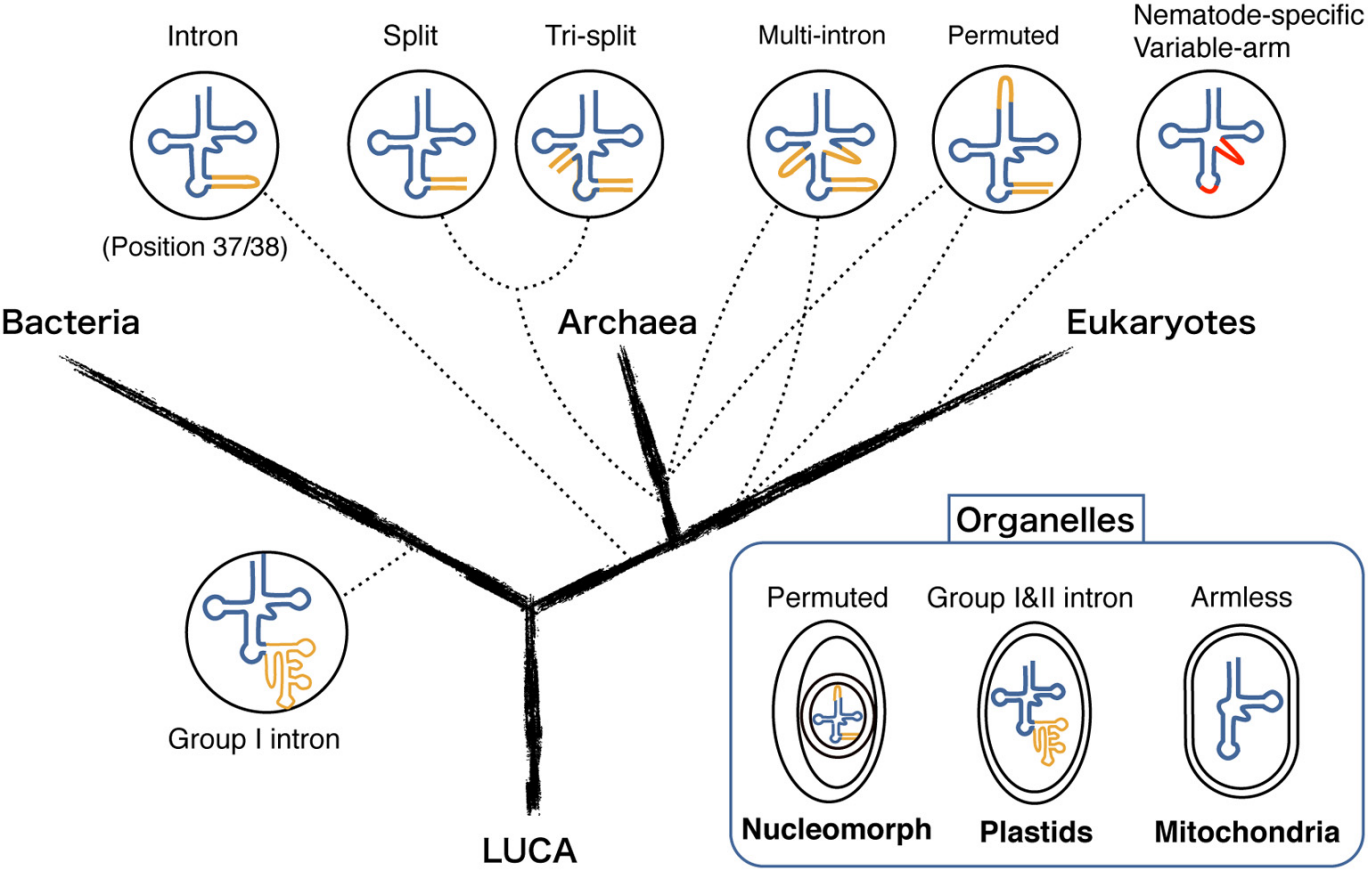
**Diversity of modern tRNA genes in the three domains of life and various organelles**. Different types of tRNA genes are mapped on the phylogenetic tree of the three domains (Bacteria, Archaea, and Eukaryotes) derived from Last Universal Common Ancestor (LUCA). Dotted lines represent the location of where each tRNA gene type is found. Intron and spacer sequences are represented in orange. Regions deviating from the standard tRNA are shown in red.

In the current era of large scale-genomics a large number of complete genome sequences are available; 2615 Bacteria, 166 Archaea, 171 Eukaryote, and 3490 Virus (March 2014; https://www.ebi.ac.uk/genomes/). tRNA genes have been long predicted computationally using a classical software tRNA-scanSE which enables one to identify 99–100% of canonical cloverleaf structure and introns at the anticodon loop with very few false positives (Lowe and Eddy, 1997). However, in 2005, a first example of *trans*-spliced tRNA encoded on two separate genes, so-called split tRNA was identified (Randau et al., 2005a). Two tRNA halves are bound by the complimentary leader sequence that forms the characteristic BHB motif at the tRNA exon-intron boundary. In an attempt to find structurally disrupted tRNA genes, our team developed a new tRNA prediction software SPLITS and SPLITSX (Sugahara et al., 2006, 2007) that centers on finding multiple intron-containing tRNA and split tRNA through detection and removal of BHB motifs at the genome level. For similar reasons, improved version of tRNA-scanSE was recently launched (Chan et al., 2011). Based on these powerful computational approaches, a variety of non-standard tRNA genes have been found in all three domains of life and organelles (Table 1). We also summarized the tRNA gene diversity on the tree of life (Figure 1). Domain Archaea provides many of the examples of non-standard tRNAs such as multiple-intron containing tRNAs (Sugahara et al., 2008), split tRNAs (Randau et al., 2005a; Chan et al., 2011), tri-split tRNAs (Fujishima et al., 2009), and permuted tRNAs (Chan et al., 2011). Permuted tRNAs are also found in the nuclear and nucleomorph genomes of early-diverged eukaryotic algae (Soma et al., 2007; Maruyama et al., 2010). The only exception (exception to what?) is the Nematode-specific variable arm containing tRNAs (nev-tRNAs) that decode alternative genetic code found in the phylum Nematoda (Hamashima et al., 2012). Many metazoan mitochondrial tRNAs are known for their armless tRNA structures lacking either one, or in extreme case, both of their arms (Ohtsuki et al., 2002; Masta and Boore, 2008). On the contrary, bacterial tRNA genes are surprisingly uniform and lack structural diversity. The only significant example found so far is the insertion of group I and II introns between nucleotides 37 and 38 of the precursor tRNA (37/38) when they are predominantly positioned adjacent to the anticodon (Paquin et al., 1997; Vogel and Hess, 2001). Similarly this location has also been known as “canonical” intron insertion site for enzymatically spliced introns in the tRNA precursors of Archaea and Eukaryotes (Abelson et al., 1998).

**Table 1:** **List of various types of tRNA genes found in the three domains of life**.

**Name**	**Bacteria**	**Archaea**	**Eukaryotes Genome**	**Organelles**	**References**
Group I intron containing tRNA	Cyanobacteria, alpha-proteobacteria and beta-proteobacteria	–	–	Plastids, Chroloplast	Reinhold-Hurek and Shub, 1992; Tanner and Cech, 1996
Intron-containing tRNA (single)	–	All known Archaea	Most eukaryotes	–	Abelson et al., 1998; Marck and Grosjean, 2003
Intron-containing tRNA (multiple)	–	Crenarchaeota, *Nanoarchaeum equitans* (Nanoarchaeota), *Methanopyrus kandleri* and *Methanothermobacter thermautotrophicus* (Euryarchaeota), *Koraechaeum cryptofilim* (Korarchaeota), ARMAN-1 and 2	*Cyanidioschyzon merolae* (red alga)	–	Sugahara et al., 2006, 2008, 2009; Soma et al., 2013
Split tRNA	–	*Nanoarchaeum equitans* (Nanoarchaeota), *Aerophyrum pernix, Staphylothermus marinus, Thermosphaera aggregans and Caldivirga maquilingensis* (Crenarchaeota)	–	–	Randau et al., 2005a; Chan et al., 2011
Tri-split tRNA	–	*Caldivirga maquilingenesis* (Crenarchaeota)	–	–	Fujishima et al., 2009
Permuted tRNA	–	*Thermofilum pendens* (Crenarchaeota)	*Cyanidioschyzon merolae* (red alga), Four prasinophyte algae	Nucleomorph in *Bigelowiella natans* (green algae)	Soma et al., 2007; Maruyama et al., 2010; Chan et al., 2011
Nematode-specific V-arm-containing tRNA (nev-tRNA)	–	–	Nematodes *(Caenorhabditis elegans, C. brenneri, C. briggsae, C. japonica, C. remanei, Pristionchus pacificus, Meloidogyne incognita, M. hapla)*	–	Hamashima et al., 2012
Armless tRNA	–	–	–	Most metazoan mitochondria	Okimoto and Wolstenholme, 1990; Ohtsuki et al., 2002; Ohtsuki and Watanabe, 2007; Masta and Boore, 2008

### Intron-containing tRNA

tRNA splicing events occur in all three domains: bacteria, archaea, and eukaryotes. In bacteria, tRNA introns are self-splicing group I introns found mostly in cyanobacteria and in few alpha- and beta-proteobacteria (Reinhold-Hurek and Shub, 1992; Tanner and Cech, 1996). There are currently two group I intron families in cyanobacteria. One is found in tRNA^fMet^ that are recently recently gain by lateral gene transfer. The other one has more ancestral origin, found at exact same position of tRNA^Leu^ (UAA) genes in both cyanobacteria and organelles called plastid that originated from endosymbiotic cyanobacteria (Paquin et al., 1997). The typical secondary structure of a group I intron consists of approximately 10 helical elements with roughly 100 nucleotides as central catalytic core of the intron RNA to facilitate the splicing reaction (Haugen et al., 2005). The majority of the bacterial and plastid tRNA group I introns are located at position 37/38 immediately downstream of the anticodon. Interestingly the same feature is observed for eukaryotic and archaeal tRNA introns that are relatively short in length (less than 100 nt) and are spliced by a series of protein enzymes called splicing endonucleases (Abelson et al., 1998). Eukaryotic and archaeal tRNA splicing share similar RNA motif recognized by orthologous splicing endonucleases, and thus origin of the tRNA introns at canonical position is assumed to be very ancestral (Kanai, 2013). Whereas, recent bioinformatics studies have revealed a number of archaeal introns located at non-canonical positions and even some tRNA genes that harbor multiple introns (Sugahara et al., 2006, 2009). There is a clear trend showing that multiple intron-containing tRNAs are prominent in the archaeal order Thermoproteales. In the most extreme cases, more than half of the tRNA genes are intervened by multiple introns (Sugahara et al., 2008). Comprehensive sequence comparison of introns among seven Thermoproteales clearly show that similar intron sequences are observed among diverse tRNA species, indicating a large-scale intron transposition occurred within this archaeal order and that could have contributed to the rapid gain of introns (Fujishima et al., 2010).

### Split tRNA and tri-split tRNA

**Figure 2:**
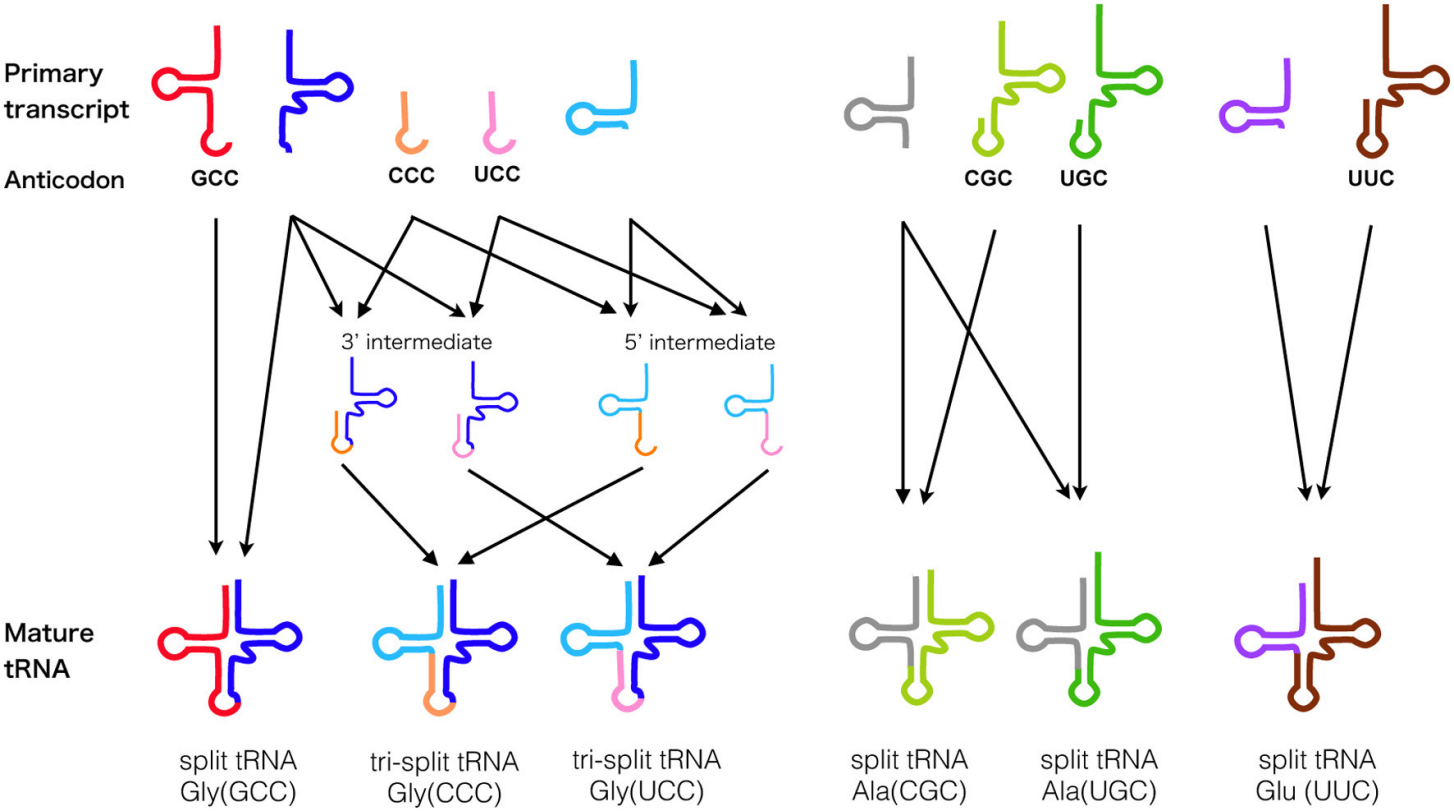
**Splicing pathway of tRNA fragments in *C. maquilingensis***. Splicing step of the ten tRNA primary transcripts that fabricate total six split/tri-split tRNAs in archaeon *C. maquilingensis*. Anticodons are denoted for the corresponding primary tRNA transcripts, and arrows represent their combination for maturation.

The first example of *trans*-spliced tRNA was reported in 2005 by Dieter Söll's group from a highly reduced genome of an ultrasmall nanoarchaeal parasite *Nanoarchaeum equitans*, termed as “split tRNA” (Randau et al., 2005a). In total, 11 fragmented tRNA genes encoding 5′ or 3′-tRNA halves were determined through expression and sequencing analysis. This also included a unique case where one 3′-tRNA^Glu^ half was shared by two 5′-tRNA^Glu^ halves (Randau et al., 2005b). tRNA halves are joined in *trans* through annealing of complimentary leader sequences and forms a relaxed BHB motif at the leader-exon boundary to be processed by the tRNA splicing endonuclease. Because of its unique features, split tRNA was first considered as a by-product of genome size reduction, however recently sequenced Nanoarchaeota Nst1 genome held no split tRNA genes, indicating that the splitting event is a specific feature of *N. equitans* and seems to reflect ongoing genome rearrangement (Podar et al., 2013). However, in 2009, our group found a new set of split tRNA genes in a free-living hyperthermophilic crenarchaeon *Caldivirga maquilingensis* genome without obvious trace of genome rearrangement nor reduction. We also found a new type of split tRNA consists of a maximum of three different RNA pieces thus coined as “tri-split tRNA” (Fujishima et al., 2009). Interestingly, some of these genes that carry anticodon sequence can be swapped with other genes to create synonymous tRNA, just like a jigsaw puzzle. In Figure 2, we summarized all the combinations of primary split/tri-split tRNA transcripts forming mature tRNAs in *C. maquilingensis*.

Split tRNA genes have also been discovered from four archaeal species belonging to the Desulfurococcales branch using an improved version of tRNA-scanSE (Chan et al., 2011). To date, total 29 trans-spliced tRNA genes corresponding to 12 anticodons have been identified in seven hyperthermophilic archaeal genomes (Table 1). The ligation sites of split tRNA gene varies, however it generally overlaps with the frequent intron insertion site of Archaea (Fujishima et al., 2010). That said, in Section Split tRNA and Intron-Containing tRNA Early or Late?, we will explain some of the possible hypotheses that explain the origin and evolution of these non-contiguous tRNAs.

### Permuted tRNA

Permuted tRNA is another form of disrupted tRNA gene where the 3′ half of the tRNA lies upstream of the 5′ half. It was first discovered in the nuclear genome of unicellular red alga *Cyanidioschyzon merolae*, (Soma et al., 2007). Expression analysis revealed that the BHB motif is formed at the termini of permuted tRNA precursor, which is further spliced and ligated into a characteristic circular tRNA intermediate. This circular intermediate is then further processed at the acceptor stem, possibly by RNase P and tRNase Z for maturation (Soma et al., 2007). SPLITS has not only contributed in finding permuted tRNA in the genome of *C. melorae* but also in other diverse photosynthetic eukaryotes including Chlorophytes, a clade of unicellular green algae, and nucleomorph genome of the green alga *Bigelowiella natans* (Maruyama et al., 2010). A nucleomorph is a vestige of primitive algal nuclei which has undergone a process known as secondary endosymbiosis. The lack of permuted tRNA in other known nucleomorph genomes and the patchy distribution of the permuted tRNA species among unicellular eukaryotes supports the gain and loss of permuted tRNA during the evolutionary stages of red and green algae (Maruyama et al., 2010). In 2011, first examples of archaeal permuted tRNA genes were found in the Crenarchaeota *Thermofilum pendens* genome, expanding the realm of permuted tRNAs to eukaryotes and Archaea (Chan et al., 2011). Given the accumulating phylogenomic evidence supporting the eocyte hypothesis, a theory where eukaryotes originate within the archaeal tree and share a sister-group with Crenarchaeota (Cox et al., 2008; Williams et al., 2013), we expect precise phylogenetic studies of permuted tRNAs and their splicing enzymes that may further strengthen this hypothesis.

### Co-evolution of splicing endonuclease and tRNA

**Figure 3:**
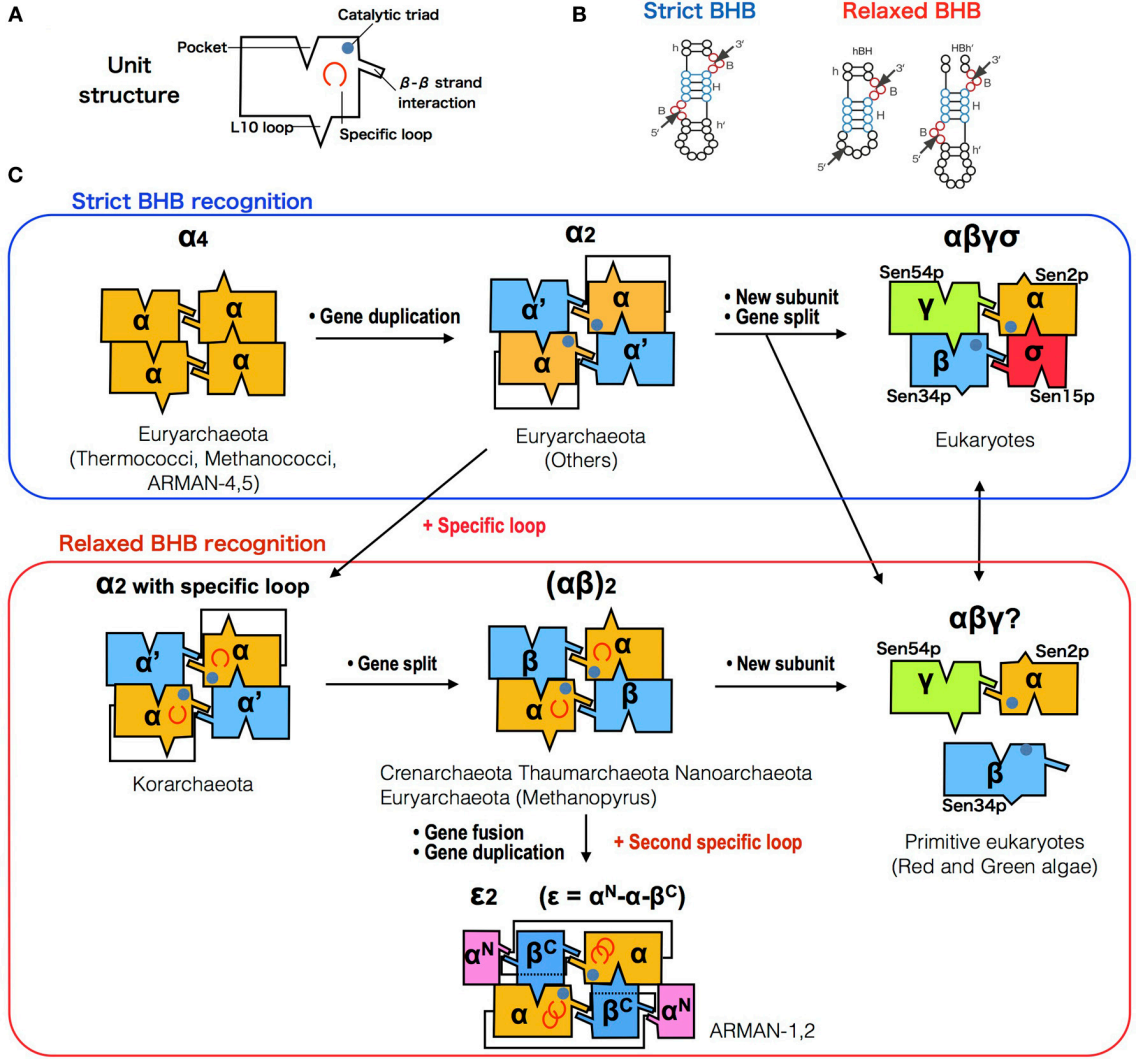
**Diversification of splicing endonuclease family and their RNA substrate specificity. (A)** Box representation of endA protein unit structure. **(B)** Comparison of strict (left) and relaxed (right) form of BHB motifs. **(C)** Possible evolutionary path of *endA* protein family based on sequence, phylogenetic (Randau et al., 2005c; Tocchini-Valentini et al., 2005b; Fujishima et al., 2011) and molecular studies (Calvin and Li, 2008; Yoshinari et al., 2009; Hirata et al., 2012; Soma et al., 2013). Unit/subunit architecture of five known types [archaeal α_4_, α_2_, (αβ)_2_, ε_2_, and eukaryotic αβγδ] and two hypothetical types (korarchaeal α_2_ with specific loop, and unknown *C. melorae* endonuclease) are shown, with colors indicating the phylogenetic relationship.

So far, the RNA splicing endonuclease *endA* family is known to be the only enzyme responsible for processing the characteristic BHB motif found in precursor sequences of tRNA (Marck and Grosjean, 2003; Sugahara et al., 2007), rRNA (Tang et al., 2002), and mRNA (Yokobori et al., 2009) in Archaea, as well as removal of tRNA introns in eukaryotes. Figure 3 represents the diversity and evolutionary events that possibly occurred during the course of *endA* gene evolution. Currently four different types of archaeal splicing endonucleases have been identified along with their crystal structures. Both homotetrameric α_4_ type and homodimeric α_2_ type in Euryarchaeota are only capable of cleaving canonical BHB motifs (Li et al., 1998; Li and Abelson, 2000). Heterotetrameric (αβ)_2_ is mostly found in Crenarchaeota (Tocchini-Valentini et al., 2005b) with a few exceptions found in Nanoarchaeota, *N. equitans*, and Euryarchaeota *Methanopyrus kandleri* (Randau et al., 2005c). Recently a fourth type of endonuclease, an unique three-unit homodimeric ε_2_ type was found in ultrasmall acidophilic archaeon ARMAN-1 and 2 (Fujishima et al., 2011; Hirata et al., 2012) and it has been shown to cleave non-canonical introns (BHL and hBH) inserted at various positions of tRNA (Tocchini-Valentini et al., 2005a; Fujishima et al., 2011). Indeed, in the crenarchaeal order thermoproteales, large numbers of tRNA introns seem to have rapidly accumulated, generating multiple intron-containing tRNAs with a maximum of three introns. This phenomenon strongly suggest that the change of splicing endonuclease type will directly influence the tRNA gene structure (Sugahara et al., 2008). In (αβ)_2_ type and ε_2_ type endonucleases, an insertion of specific loops were observed and based on the structural analysis, both loops are essential for the recognition of relaxed BHB splicing motif found at the boundaries of tRNA exon and non-canonical introns (Hirata et al., 2012). These results clearly show the evolutionary path of splicing endonuclease toward acquiring broad substrate specificity, which drove the co-evolution along with tRNA genes to accept introns at various positions. Based on the sequence evidence and phylogeny of subunits, we recently proposed a novel homodimeric α_2_ type of archaeal endA specifically found in Korarchaeota, which shares a same “specific loop” with the (αβ)_2_ type, known to be essential for the cleavage of non-canonical tRNA introns (Fujishima et al., 2011). Indeed, *Korarchaeum cryptofilum* is the only archaeon with α_2_ endonuclease that harbors tRNA genes with non-canonical introns, representing another example of the tRNA—*endA* co-evolution. In eukaryotes, tRNA splicing endonuclease consists of four subunits Sen2p, Sen15p, Sen34p, and Sen54p, in which Sen2p and Sen34p share clear sequence homology to archaeal endA (Trotta et al., 1997). The function of eukaryotic endA has been well studied in yeast, where the yeast splicing endonuclease can only strictly cleave tRNA introns located at position 37/38 just after the anticodon (Reyes and Abelson, 1988). Highly conserved 50 aa core sequence shared between the two domains of life suggest a monophyletic origin of RNA splicing endonuclease as a single subunit protein forming a homotetramer known as α_4_ type, emerged before the divergence of Archaea and eukaryotes (Abelson et al., 1998). While protein crystal structures of the four types of archaeal endonucleases have been solved, so far only one of the four eukaryotic endA subunits sen15p has been crystalized from human homolog (Song and Markley, 2007), leaving overall orientation of the eukaryotic tRNA splicing endonuclease up to speculation. Two-hybrid experiment has previously shown that *in vivo*, interaction occurs between Sen2p and Sen54p, and between Sen34p and Sen15p specifically, leading to a heterotetrameric enzyme model with Sen54p functioning as a ruler to measure the distance from the tRNA mature domain (Trotta et al., 1997). However, the genome sequence of primitive red algae *C. melorae* and green algae harbors highly-disrupted tRNA genes including permuted tRNA and multiple intron-containing tRNA. Only three homologs, cmSen2p, cmSen34p, and cmSen54p have been identified from the *C. melorae* genome (Tocchini-Valentini and Tocchini-Valentini, 2012). More recently, a yeast-two hybrid experiment revealed a reciprocal interaction between cmSen2 and cmSen54, however cmSen34p did not interact with either of the two subunits suggesting a distinct complex formation and splicing machinery from canonical eukaryotic heterotetramer (Soma et al., 2013).

### Nev-tRNA (nematode-specific V-arm-containing tRNAs)

Mature tRNA can be grouped into two distinct classes (I and II) based on the presence of the long variable arm (V-arm) located between the anticodon arm and the T-arm (Brennan and Sundaralingam, 1976). This V-arm has known to be specific for tRNA^Ser^, tRNA^Leu^, and bacterial tRNA^Tyr^, and it plays an important role in the recognition of cognate aminoacyl tRNA synthetase (aaRS) (Tocchini-Valentini et al., 2000). However, comprehensive analysis of 46 diverse eukaryotic genomes revealed over 100 novel class II tRNAs in six nematodes genomes with a non-canonical anticodon, coined as nematode-specific V-arm-containing tRNA (nev-tRNA) (Hamashima et al., 2012). Comparative sequence analysis of nev-tRNA has shown that aaRS recognition elements in the V-arm are similar to that of class II tRNA. Indeed, aminoacylation assay and *in vivo* translation experiment have shown that nev-tRNA^Gly^ can decode Gly(GGG) codon as leucine. Expression levels of two nev-tRNA^Gly^(CCC) and nev-tRNA^Ile^(UAU) turned out to be very low, compared to that of canonical tRNAs in *C. elegans*. Moreover, codons that nev-tRNAs correspond to are mostly rare codons and comprise less than 20% of the synonymous codons, suggesting it will not drastically influence the proteome (Hamashima et al., 2012). Recently we have identified additional nev-tRNAs in two plant-parasitic nematodes belonging to genus *Meloidogyne*. Similar anticodon alternation has been reported in other organisms including primates, *Drosophila*, yeast, and Enterobacteria, in which half of the cases were involved in switching the codon identity (Rogers and Griffiths-Jones, 2014). In extreme cases, anticodon shift results in assigning an alternative genetic code in certain species, such as the stop codons UGA or UAA being reassigned to various sense codons in *Mycoplasma*, certain ciliated protozoans, and peritrich species (Hamashima and Kanai, 2013).

### Mitochondrial armless tRNA

Mitochondria are an essential organelle in eukaryotes, generating most of the cell's energy supply as ATP. It is generally accepted that mitochondria originated from within the bacterial phylum α-Proteobacteria and underwent an endosymbiotic event (Gray, 2012). With few exceptions, animal mitochondrial genomes carry over 20 tRNA genes that are distinct from their host cell tRNA (Boore, 1999). However, some nucleus-encoded tRNAs have to be imported from the cytosol to complete the mitochondrial translation (Salinas et al., 2012). Mitochondria have also developed their own variant genetic codes from the universal genetic code, repeatedly and independently in various eukaryotic taxa (Knight et al., 2001). Currently a compilation of over 30,525 mitochondrial tRNA (mt tRNA) sequences from 1418 fully sequenced metazoan mitochondrial RefSeq genomes are registered in the mitotRNAdb (http://mttrna.bioinf.uni-leipzig.de) (Jühling et al., 2009). Most mt tRNAs possess a canonical cloverleaf structure, however extreme examples of truncated mt tRNAs have been identified in some metazoan mitochondria (Ohtsuki et al., 2002; Masta and Boore, 2008). D-armless and T-armless mt tRNAs were first identified in two nematode worms *C. elegans* and *Ascaris summ* (Okimoto and Wolstenholme, 1990). Later, a significant number of T-armless mt tRNAs were found in six different eumetazoa phylum including Nematodes and Arthropods (Ohtsuki and Watanabe, 2007). On the contrary, mammalian mitochondria possess only the cloverleaf and D-armless tRNA, and so far truncated mt tRNA have not been observed in either plants or fungi, suggesting that D-armless tRNA first emerged after the branching of metazoa and more recently T-armless mt tRNA arose in independent branches of eumetazoa (Ohtsuki and Watanabe, 2007). It has been speculated that the main cause of such diversification is due to the subfunctionalization of elongation factor EF-Tu. In *C. elegans*, mitochondrial DNA encodes two EF-Tu homologs, EF-Tu1 and EF-Tu2, which exclusively recognize aminoacylated T-armless and D-armless mt tRNAs, respectively (Arita et al., 2006). Given the fact that in mammalian mitochondria, cloverleaf and D-armless tRNAs are recognized by a single mt EF-Tu that resembles bacterial type (Andersen et al., 2000), gene duplication of EF-Tu leading to a subfunctionalizion of EF-Tu1 to recognize T-armless mt RNA should have contributed in the co-evolution of mt tRNA species, allowing their extreme truncation (Arita et al., 2006).

## Origin and evolution of tRNA—molecular evidence and evolutionary scenario

tRNA is unique for its capability to bridge information from nucleotide polymer (RNA) to amino acid polymer (protein). Decoding of mRNA information by tRNA is governed by triplet codon-anticodon base pairing, and each codon corresponds to one of the 20 standard proteinogenic amino acids that all living organism share in common. These amino acids are correctly charged onto the 3′-adenosine terminal of the tRNA molecule by aaRSs. Currently two evolutionary unrelated classes of tRNA synthetases (Class I and Class II) are known (Wolf et al., 1999), and surprisingly for many of these aaRSs, anticodon arm is not necessary for aminoacylation, capable of charging short RNA minihelices and duplexes that including the minimal 12 bp acceptor arm-TΨC stem loop, known to be the top half of tRNA (Francklyn and Schimmel, 1989, 1990). This finding led to the theory of “operational code,” which in the early stage of tRNA evolution, identity was solely embedded in the simple minihelix RNA which later became the acceptor stem of tRNA (Schimmel et al., 1993). Hence, in this chapter, we will feature major findings and theories that lead to a plausible scenario for the origin and evolution of tRNA molecule.

### Minimal aminoacyl ribozyme

**Figure 4:**
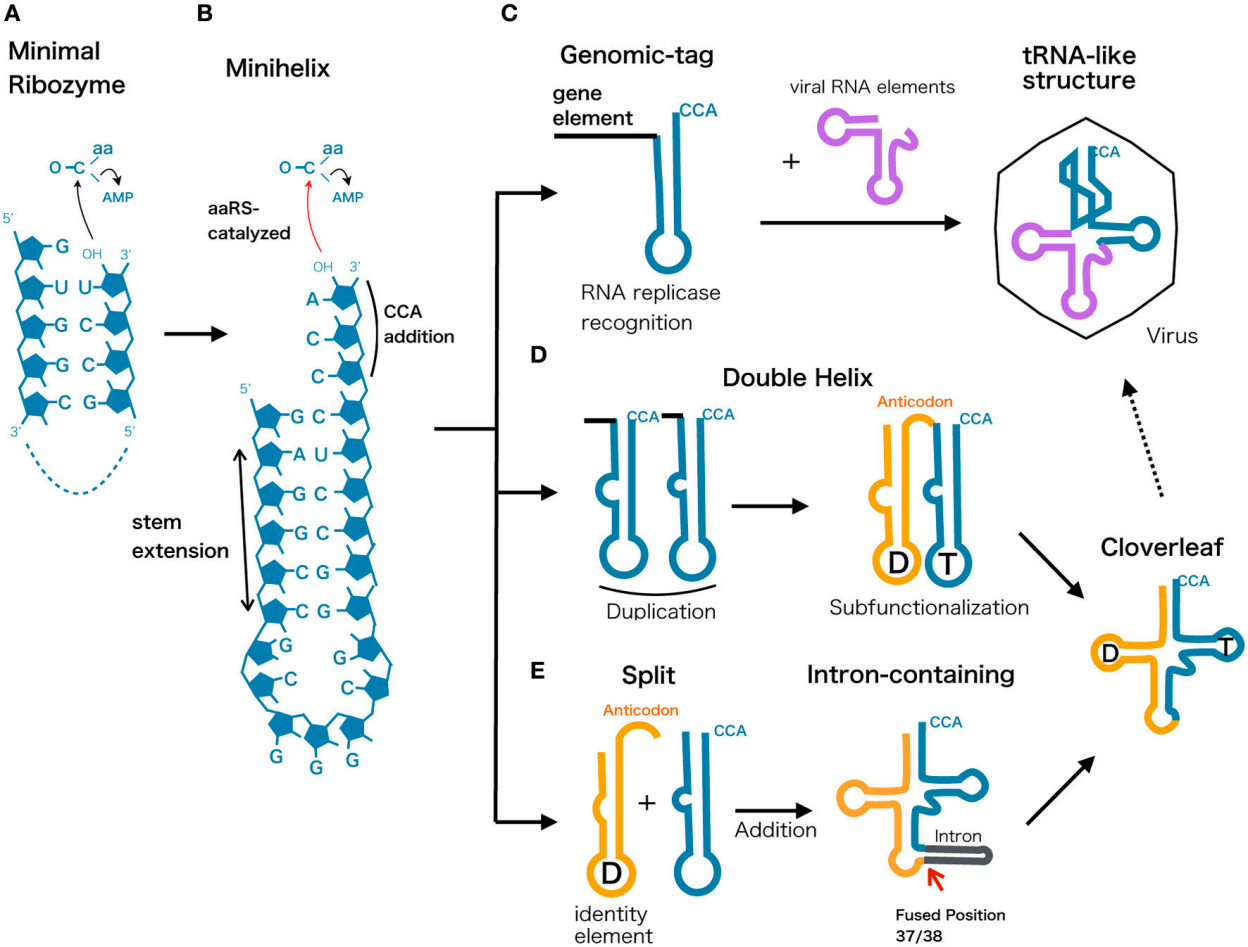
**Possible evolutionary scenarios from ancestral ribozyme to modern tRNA**. The evolutionary scenarios of tRNA molecule represented along with proposed primordial models. **(A)** Minimal ribozyme that catalyzes and generates short peptidyl-RNA, **(B)** minihelix harboring the 3′-CCA terminal sequence, **(C)** Genomic-tag hypothesis showing the recognition of minihelix by RNA replicase. This functionality could have been retained and integrated into the tRNA-like structure found in modern viral genomes. **(D)** Double helix model assuming a duplication event that led to a subfunctionalization of two hairpin loops that are later diversified into D-arm and anticodon plus T-arm. **(E)** Split tRNA early model, showing a fusion of different minihelices that eventually led to modern cloverleaf. This is based on the fact that tRNA introns are universally and predominantly found at position 37/38 and that intron could be a reminiscent scar of gene fusion.

The essentiality of tRNA lies in its core function of 2′-3′ aminoacylation. Modern aaRS catalyze this reaction as a two-step reaction (1) Amino acid activated by ATP, forming an intermediate aminoacyl adenylate (aa-AMP), (2) Aminoacyl group is transferred from the adenylate to the tRNA 3′-terminal adenosine nucleotide. However, modern aaRSs are the product of aminoacylation-based translation and thus researchers have been seeking for a more primitive chemical path that eventually led to this sophisticated machinery. For example, it has been shown that high energy aminoacyl adenylate can be formed from ATP and amino acids under prebiotic conditions (Paecht-Horowitz and Katchalsky, 1973). Furthermore, an *in vitro* evolution experiment has achieved in selecting a ribozyme capable of self-aminoacylating its own 5′-hydroxyl group and transferring the aminoacyl group to the 3′-end of other RNA, supporting the idea that aminoacyl-tRNA synthetase ribozymes playing a important role in the RNA world (Lee et al., 2000). The most extreme example of aminoacylating ribozyme to date, is a 5-nt ribozyme discovered though radical minimization of C3 ribozyme that self-aminoacylates (Turk et al., 2010), this ultrasmall ribozyme initially trans-phenylalanylates a complementary 4-nt RNA selectively (Figure 4A). The transfer reaction occurs regiospecifically from the phenylalanine atom to ribose 2′-hydroxyl, forming an ester bond between amino acid and RNA that is identical to the modern aminoacylated tRNA. This discovery implies that short ribonucleotides can aminoacylate its counterpart to produce peptidyl-RNA, which can be interpreted as the minimal form of tRNA that could have originated in the very early stage of life.

### Minihelix model

One of the plausible models for the early stage of tRNA evolution is known as a minihelix or a minigene that forms a hairpin structure (Figure 4B). This concept comes from the fact that L-shaped tertiary structure can be distinguished into two halves, where only the top part of tRNA (acceptor stem + TΨC arm) is recognized by many of the modern tRNA synthetases (Schimmel and Ribas de Pouplana, 1995). The identity of tRNA is still embedded within the acceptor stem, generally depending on position discriminator base N73 and few base pairs downstream, termed the operational RNA code (Schimmel et al., 1993). Along with the discovery of minimal self-aminoacylating ribozymes presented in the previous section, presumably aminoacylated minihelix originated in an environment where short RNA oligos and amino acids co-existed, prior to the emergence of the ribosome. Tamura and Schimmel demonstrated that D-ribose chirality of RNA minihelix exhibited a clear preference for charging L-amino acids as opposed to D-amino acids (Tamura and Schimmel, 2004), indicating that aminoacylation of RNA could have been the key toward modern protein homochirality. Consequently, ribozyme-based aminoacylation was eventually substituted by more efficient and less promiscuous homochiral protein-based aminoacylation.

### Genomic-tag hypothesis and tRNA-like structure

The top half of modern tRNAs is recognized by RNase P, CCA-adding enzyme, and tRNA synthetases which are all related to the maturation of tRNA molecules function in protein synthesis. Various positive strand RNA viruses in plants possess tRNA-like structures (TLSs) at the 3′-end of their genome sequences that are functional mimics of tRNA (Dreher, 2009) (Figure 4C). They fall into three types, mimicking the tRNA function of specific aminoacylation by valine, histidine, or tyrosine at the 3′ end of pseudoknotted aminoacyl acceptor stem (Pleij et al., 1985). Surprisingly their roles are diverse; tRNA mimicry providing translational enhancement, presentation of minus strand promoter elements for RNA replicase recognition, recruitment of host CCA nucleotidyltransferase as a 3′-telomere, and *in vitro* packaging of the viral genome (Dreher, 2010). Similar trend in using TLS for replicator element can also be seen for most retroviral RNA genomes and long terminal repeat (LTR)-retrotransposons, in which annealing of the top half tRNA to the primer binding site initiates the reverse transcription (Le Grice, 2003). The growing evidence of tRNA elements involved in RNA and DNA replication has therefore led to the idea of the “Genomic Tag Hypothesis” noting that tRNA-like structural motifs initially evolved as a 3′ terminal motif that tagged RNA genomes for replication in the RNA world before the advent of protein synthesis (Weiner and Maizels, 1999).

### Double helix model

From a thermodynamic perspective, modern cloverleaf structure of tRNA is not a minimum free energy state but rather a local minimum structure that is reinforced by multiple post transcriptional modification (Wuchty et al., 1999). Similarities between nucleotides at comparable positions within the 5′ and 3′ halves of the tRNA molecule have often been taken as evidence that the modern cloverleaf structure arose through direct duplication of a hairpin (Tanaka and Kikuchi, 2001; Widmann et al., 2005) (Figure 4D). Indeed, one of the suboptimal structures of tRNA is a double helix structure that closely resembles a duplicate form of a minihelix and this structural feature was confirmed by *in vitro* cleavage of tRNA using RNase P (Hori et al., 2000). One possible scenario is that after the duplication event, the latter top half with 3′-CCA terminal continued its role as a recognition RNA element, while bottom half underwent subfunctionalization to provide anticodon and sequence diversity to coevolve with variants of tRNA synthetases.

### Split tRNA and intron-containing tRNA early or late?

The symmetric form of tRNA is also considered as a consequence of two separate hairpin RNAs fused to form a modern tRNA structure (Figure 4E). The evidence showing that intron sequence is universally found at position 37/38 just after the anticodon indicates a scenario that introns could be a trace of the assembled two hairpin RNA genes (Di Giulio, 2006). The difference from the duplicated double helix model is that the later added bottom half of the tRNA (D-arm + anticodon) could be evolutionary distinct from the top half. We have previously suggested the possibility of separate origin of 5′ and 3′ tRNA halves based on the sequence similarity and diversity in archeal tRNAs (Fujishima et al., 2008). Furthermore, based on phylogenetic studies of both structural and statistical characteristics of over 500 tRNA molecules suggests that the bottom half of the tRNA was added later in time (between 3 to 4 billion years ago) to the ancient top half, which grew by slow substructural accretion (Sun and Caetano-Anollés, 2007). Currently, split tRNA genes that separately encode the 5′ and 3′ halves of tRNAs are found in several archaeal genomes (see Section Split tRNA and Tri-Split tRNA for detail). Their split positions and flanking sequences resemble the intron in related species, indicating a strong evolutionary link between split tRNA and intron-containing tRNA (Fujishima et al., 2008, 2009). It is likely that the split tRNA that we currently see in several archaeal genomes is a recently acquired trait. For example, recently the sequenced genome of a first terrestrial hyperthermophilic member of nanoarchaeota Nst1 possess not even a single split tRNA gene (Podar et al., 2013). Interestingly, while *N. equitans* possess (αβ)_2_ type endonucleases capable of processing non-canonical introns and split tRNA, Nst1 genome encodes α_4_ type that can only cleave canonical introns. Accordingly, acquisition of (αβ)_2_ type may have allowed tRNA gene fragmentation to occur and linger in *N. equitans* genome (Podar et al., 2013). Indeed, we have recently found evidence of ongoing tRNA gene disruption from a community genomic library prepared from a *Caldiarchaeum subterraneum*-dominated microbial mat (Sugahara et al., 2012). A heterogenic clone library revealed a putative DNA recombinase coding a 1.8 kb DNA insertion separating the tRNA fragment and the tRNA gene, a feature that is frequently found at the integration site of mobile elements, such as conjugative plasmids and viruses. Randau and Söll have earlier suggested that split tRNA genes present a strategy for impeding the viral integration or insertion of other mobile elements into canonical tRNA genes (Randau and Söll, 2008). A similar conclusion was reached by Chen et al., based on the fact that 5′ and 3′ halves of pre-tRNA^Asp^(GUC) are located adjacent to each other in two related archaea *A. pernix* and *T. aggregans*, indicating two different local genome rearrangement events that occur at the same position in the same tRNA (Chan et al., 2011). On the other hand, Di Giulio interpreted this evidence from a different perspective that the current split genes we see in Archaea may represent the transition stage through which the evolution of tRNA molecule have passed (Di Giulio, 2009). He also explains that the discrepancy of having interruption at non-canonical position of some split tRNA is expected, and compatible with the split early model where assembly of the two hairpin-like structures may also have built structures to tinker with cloverleaf structure (Di Giulio, 2012).

## Conclusions

In this review, we have recapitulated the diversity of gene orientation and RNA structures of modern tRNAs in three domains of life, organelles and viruses. We also focused on the co-evolution of tRNA and their splicing endonucleases, and discussed how subfunctionalization of the enzyme could shape tRNA gene arrangement by allowing the tRNA gene to accept introns at various positions as well as allowing gene fragmentation and permutation. The origin of the tRNA intron is still under debate since intron located at canonical position 37/38 is conserved between Archaea and Eukaryotes and thus represents an ancestral trait, while non-canonical introns are likely added recently due to the change in the functionality of RNA splicing endonuclease. Canonical positioning is a plausible explanation of the hypothesis that modern tRNA arose from simple minihelix RNA through gene duplication/fusion. There is also growing evidence of virus or transposable elements involved in the recent addition of the tRNA intron. It will be important to collect snapshots of the tRNA intron insertion event through sequence analysis to reveal the precise molecular mechanism. Lastly, the charm of tRNA research lies in its potential of shaping the genetic code. There are examples of re-coding in mitochondria and higher eukaryotes possibly driven by tRNA gene multiplication, which tends to target the rare codons to avoid impact on proteome. Further accumulation of genomic and transcriptomic sequence data will likely provide further examples of modern tRNA diversity as well as insight into the origin and evolution of this essential molecule that is deeply involved in the rise of modern genetic system.

### Conflict of interest statement

The authors declare that the research was conducted in the absence of any commercial or financial relationships that could be construed as a potential conflict of interest.
